# An Electric Field Microsensor with Mutual Shielding Electrodes

**DOI:** 10.3390/mi12040360

**Published:** 2021-03-26

**Authors:** Hucheng Lei, Shanhong Xia, Zhaozhi Chu, Biyun Ling, Chunrong Peng, Zhouwei Zhang, Jun Liu, Wei Zhang

**Affiliations:** 1 State Key Laboratory of Transducer Technology, Aerospace Information Research Institute, Chinese Academy of Sciences, Beijing 100094, China; leihucheng16@mails.ucas.ac.cn (H.L.); crpeng@mail.ie.ac.cn (C.P.); zhangzhouwei15@mails.ucas.ac.cn (Z.Z.); sushi_1220@163.com (J.L.); zhangwei178@mails.ucas.ac.cn (W.Z.); 2 School of Electronic, Electrical and Communication Engineering, University of Chinese Academy of Sciences, Beijing 100049, China; 3 Institute of Microelectronics of Chinese Academy of Sciences, Beijing 100029, China; chuzhaozhi@ime.ac.cn; 4 Institute of Microsystem and Information Technology, Chinese Academy of Sciences, Shanghai 200032, China; lingbiyun@mail.sim.ac.cn

**Keywords:** electric field microsensor, mutual shielding electrodes, piezoelectric driven

## Abstract

This paper proposes an electric field microsensor (EFM) with mutual shielding electrodes. Based on the charge-induction principle, the EFM consists of fixed electrodes and piezoelectric-driving vertically-movable electrodes. All the fixed electrodes and movable electrodes work as both sensing electrodes and shielding electrodes. In other words, all the fixed and movable electrodes are sensing electrodes, and they are mutually shielding electrodes simultaneously. The movable electrodes are driven to periodically modulate the electric field distribution at themselves and the fixed electrodes, and the induced currents from both movable and fixed electrodes are generated simultaneously. The electrode structure adopts an interdigital structure, and the EFM has been simulated by finite element methods. Simulation results show that, since the sensing area of this EFM is doubled, the variation of induced charge is twice, and therefore the output signal of the sensor is increased. The piezoelectric material, lead zirconate titanate (PZT), is prepared by the sol–gel method, and the microsensor chip is fabricated.

## 1. Introduction

Electric field sensors (EFSs) have a wide range of applications in many fields [1,2,3,4,5,6,7,8,9,10,11], such as aerospace, meteorology, power systems, etc. In the aerospace field, a spacecraft needs to have good launch conditions to ensure it is launched. The value of the electric field strength is listed as one of the important conditions for the launch of a spacecraft. The electric field in the air is the direct cause of lightning. When lightning occurs, on the one hand, it may damage the outer surface of the spacecraft and affect the flight of the spacecraft; on the other hand, the lightning may damage the electronic components of the spacecraft and affect the normal operation of the spacecraft. EFSs are used to monitor the atmospheric electric field before the flight to ensure the safety of the spacecraft during launch. In the field of meteorology [4,5,6,7], different weather conditions have different corresponding atmospheric electric field values. EFSs can be used to analyze the changing characteristics and laws of the atmospheric electric field under different weather to realize the monitoring and warning of lightning weather. In the field of power systems, EFSs can realize non-contact voltage monitoring [8]. In addition, EFSs can also be applied to insulator defect detection [9], icing thickness detection on the surface of transmission lines [10], and electromagnetic environment detection around power systems [11], etc.

In the recent three decades, with the development of micromachining technology, a variety of EFMs have been reported [12,13,14,15,16,17,18,19,20,21,22,23,24,25,26,27,28,29,30], which have advantages of small volume, batch producibility and low power consumption. Most of them are charge–induction-based ones, whose sensing structures mainly consist of fixed sensing electrodes and grounded movable shielding electrodes. The movable electrodes are driven to periodically modulate the electric field distribution at the fixed sensing electrodes, and the induced currents from fixed electrodes are generated simultaneously. At present, EFMs are still difficult to meet the needs of extremely low-intensity electric field detection, and improving the resolution and sensitivity is a key issue for the research of electric field sensors.

This paper proposes an EFM with mutual shielding electrodes to improve induction efficiency, aiming at improving the sensitivity of the sensor. Electrodes in this EFM work as both sensing electrodes and shielding electrodes, and the movable electrodes are vertically driven in a piezoelectric way.

## 2. Sensor Design

### 2.1. Structure Design

**Figure 1:**
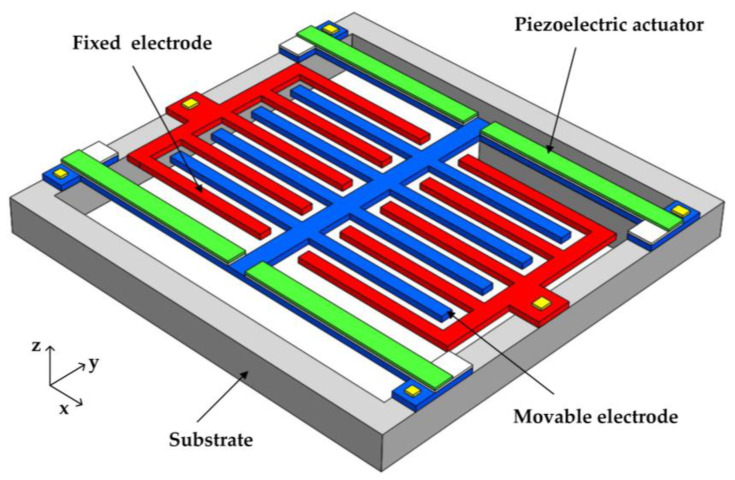
Schematic view of electric field microsensor (EFM) structure.

The schematic structure of the sensor is shown in Figure 1. The sensor is composed of fixed electrodes, movable electrodes, piezoelectric actuators and substrate. The fixed electrodes and the movable electrodes are in the same plane in the stationary state, and the fixed electrodes and the movable electrodes work as mutual shielding electrodes during the vibration of the movable electrodes. The sensor is actuated by piezoelectric actuators and vibrates vertically during operation. In Figure 1, vertical vibration refers to the vibration of the movable electrodes along the z-direction of the coordinate system. A fixed electrode and a movable electrode work as a pair of mutual shielding electrodes. The key parameters of the proposed EFM are listed in Table 1.

### 2.2. Working Principle

**Figure 2:**
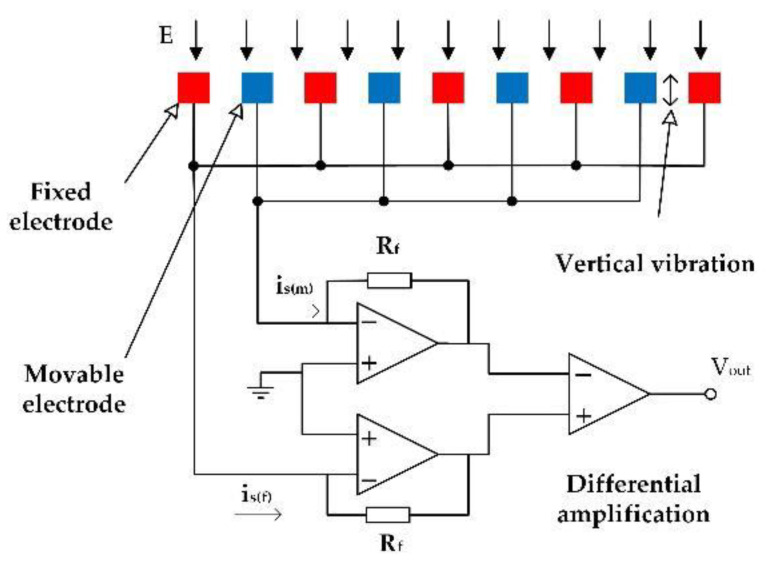
Working principle of the EFM.

The working principle of the EFM is shown in Figure 2. All the fixed electrodes and movable electrodes work as both sensing electrodes and shielding electrodes. In other words, all the fixed and movable electrodes are sensing electrodes, and they are mutually shielding electrodes simultaneously. 

When an applied electric field E reaches the sensor sensitive structure vertically, according to Gauss’s law, the corresponding induced charge is generated on the sensing electrode, and the charge quantity (Q) is given by

(1) $$Q=\varepsilon_{0}EA,$$

where $\varepsilon_{0}$ is the permittivity of free space, A is the effective area of the electrodes.

When the movable electrodes vibrate upward, electric field distribution at the fixed electrodes is weakened, and the quantity of induced charges on the fixed electrodes decreases, the movable electrodes have an enhanced charge-induced shielding effect on the fixed electrodes. Conversely, when the movable electrodes vibrate downward, the electric field distribution at the movable electrodes is weakened, the quantity of induced charges on the movable electrodes decreases, and the fixed electrodes have an enhanced charge-induced shielding effect on the movable electrodes. During the vibration of the movable electrodes, the fixed electrodes and the movable electrodes work as mutual shielding electrodes. 

The sensor is actuated by piezoelectric actuators and the movable electrodes vibrate vertically during operation to periodically modulate the electric field distribution at the movable electrodes as well as the fixed electrodes, and induced current $i_{s}$ generated on the electrodes, $i_{s}$, is given by

(2) $$i_{s}=\frac{dQ}{dt}=\varepsilon_{0}E\frac{dA}{dt}$$

The amplitude of the induced current implies the amplitude information of the applied electric field. During the vertical vibration of the movable electrodes, when the variation of induced charge on the movable electrode increases, the variation of induced charge on the fixed electrode decreases, and the variation of induced charge on the fixed electrodes and the movable electrodes forms a differential output. Induced currents generated on all the electrodes are measured by an external differential amplifier circuit.

### 2.3. Electrode Design

**Figure 3:**
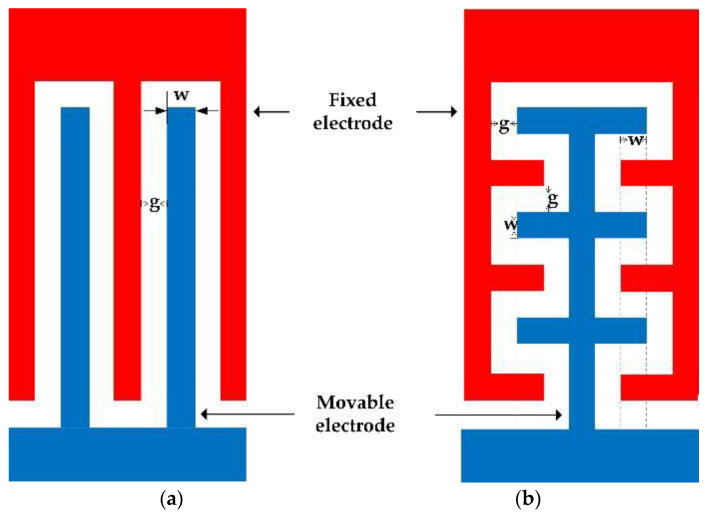
Electrodes setting. (**a**) Interdigital structure. (**b**) Comb-shaped structure.

There are two normal ways to set the electrodes, the interdigital structure and the comb-shaped structure, as shown in Figure 3.

It is assumed that the fixed and movable electrodes have the same width(w), and the gap(g) between them is equal to the width. When the total area is constant, the sensing area of the two electrode structures is calculated to be the same. According to Gauss’s law, when the total area and electric field flux are constant, the structure with a large sensing area of the electrodes obtains more electric field distributions. The electrode structure with a larger sensing area has a stronger ability to induce charges under the same conditions. Considering the complexity and controllability of the manufacturing process, the electrode structure adopts an interdigital structure.

## 3. Simulation

### 3.1. Simulation Model

**Figure 4:**
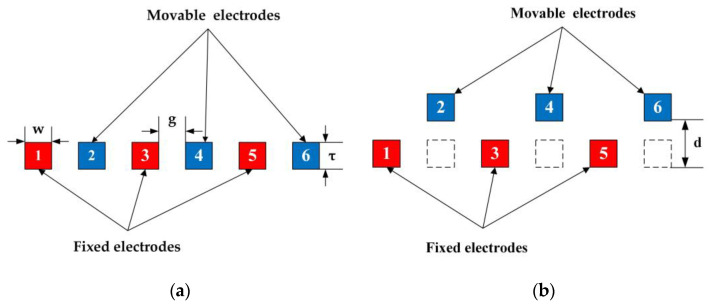
Simulation model. (**a**) Stationary state. (**b**) Moving state.

The EFM has been simulated by finite element methods, and the simulation model is shown in Figure 4. Three pairs of movable electrodes and fixed electrodes are set in the simulation model. d is the displacement of the movable electrodes, and the fixed and movable electrodes have the same thickness (τ). 

The simulations in this paper take electrode 3 and electrode 4 as examples and the applied electric field strength is 1 kV/m.

### 3.2. Simulation Results

#### 3.2.1. Electrode Width and Electrode Gap

The size of the electrode width and gap will affect the number of mutual shielding electrode groups in a certain area. When the electrode width or the electrode gap decreases, the number of mutual shielding electrode groups increases in a certain area. This paper uses parameter B [30] to illustrate the simulation results, B is given by

(3) $$B=\frac{\Delta Q}{2g+2w}.$$

ΔQ is the difference of induced charge variations between a pair of mutual shielding electrodes. In the case of a certain area, the larger the value of B is, the stronger the charge induction ability of the corresponding electrode structure will get.

**Figure 5:**
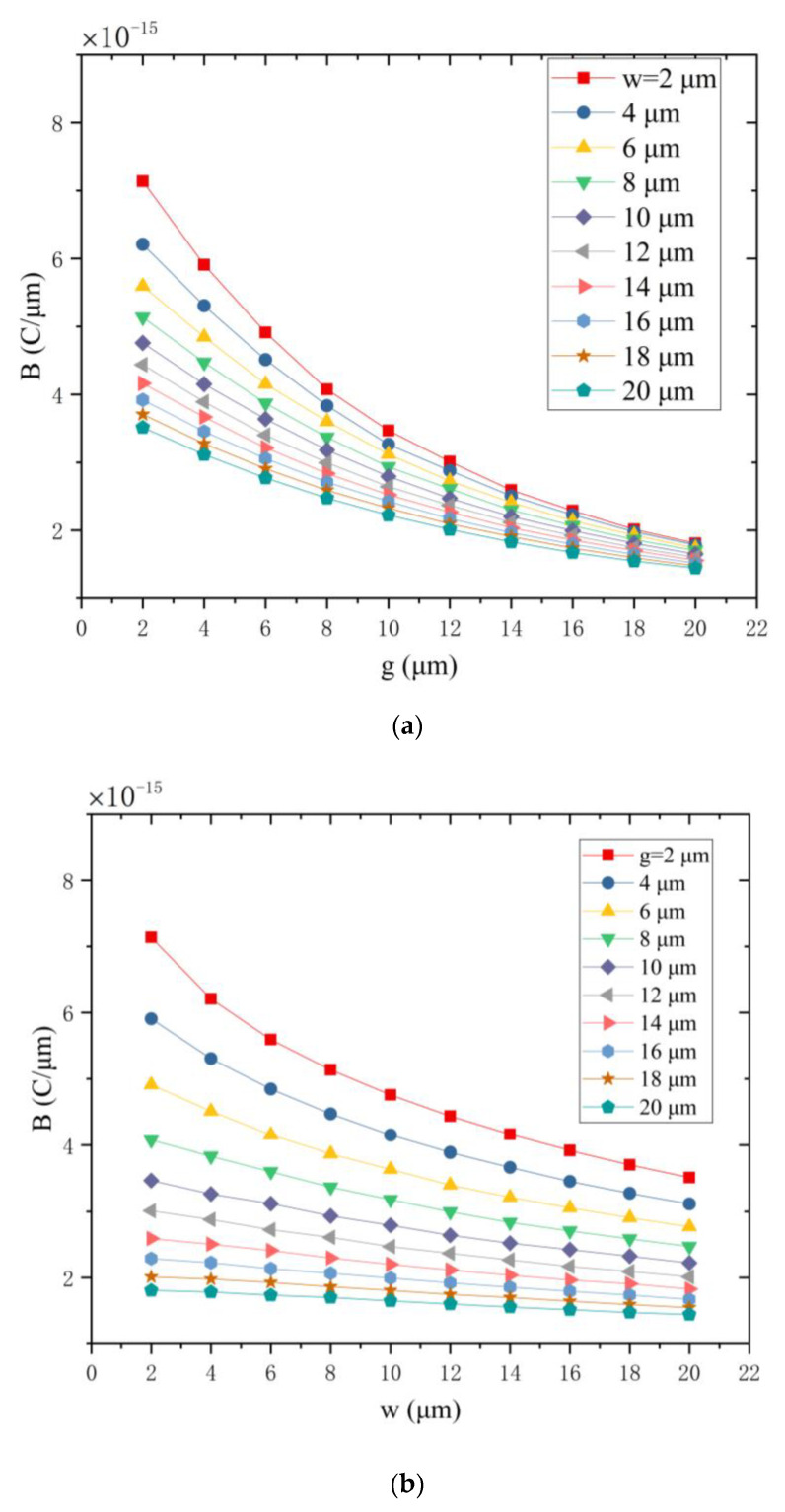
The relationship among parameter B, g and w. (**a**) The parameter B versus the electrode gap(g) under different electrode widths. (**b**) The parameter B versus the electrode width (w) under different electrode gaps.

When d is set to 10 μm and τ is set to 5 μm, by changing the size of g and w, the simulation obtains the relationship among parameters B, g and w, as shown in Figure 5. 

It can be seen from Figure 5a that as the electrode gap increases, the parameter B decreases. It can be seen from Figure 5b that as the electrode width increases, parameter B decreases. Therefore, the charge induction ability of electrodes becomes stronger as the electrode gap or the electrode width decreases.

#### 3.2.2. Electric Field Distribution

**Figure 6:**
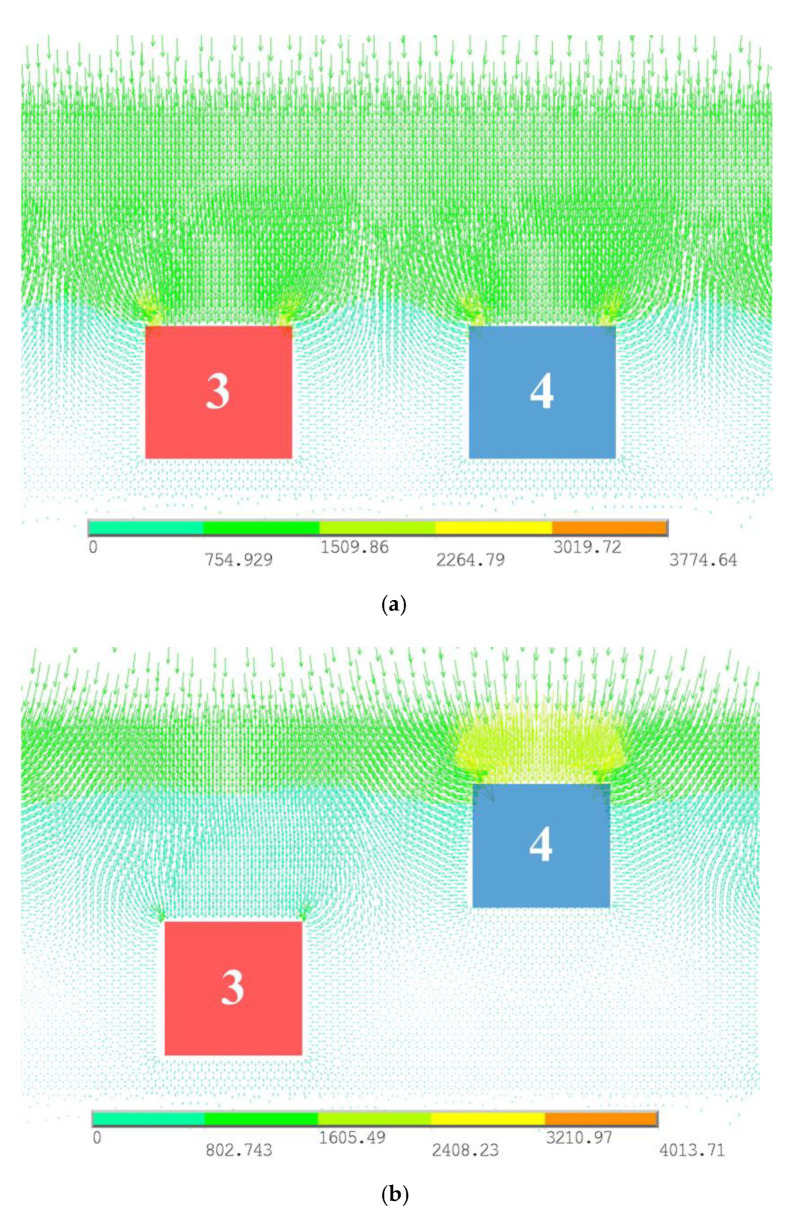
Vector-graph of electric field distribution at the electrodes. (**a**) Stationary state. (**b**) Moving state.

Considering the accuracy of the MEMS process in the laboratory, g and w are both set to 5 μm. According to the above parameters, the electric field distribution at the electrodes is simulated, as shown in Figure 6. Figure 6a shows the stationary state in which d is set to 0 μm and Figure 6b shows the moving state in which d is set to 5 μm.

As can be seen from Figure 6, when the movable electrodes vibrate upward, the electric field distribution at the fixed electrodes is weakened, while the field distribution at the movable electrodes is enhanced.

#### 3.2.3. Variation of Induced Charge

**Figure 7:**
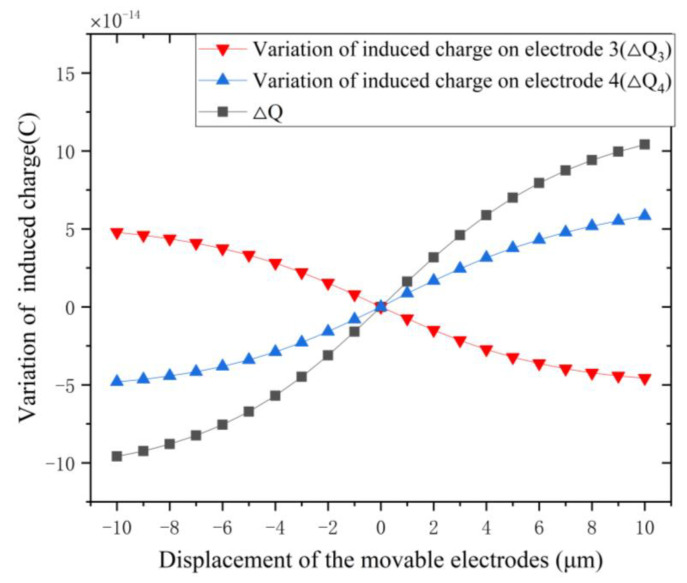
The variation of induced charge on electrodes versus the displacement of the movable electrodes.

The variation of induced charge versus the displacement of the movable electrodes is shown in Figure 7, and data of induced charge variations on electrodes are shown in Table 2. ΔQ is the difference of induced charge variations between a pair of electrodes (electrode 3 and electrode 4), assuming that the movable electrodes vibrate upward as a positive direction. The fixed electrode 3 and the movable electrode 4 work as a pair of mutual shielding electrodes.

From Figure 7 and Table 2, it is seen clearly that the variation of induced charge on a pair of electrodes (ΔQ) is about twice as much as that of a single electrode (ΔQ3), which means that, compared with previous EFMs with grounded shielding electrodes, the output signal of our EFM with mutual shielding electrodes is almost doubled.

## 4. Fabrication

### 4.1. Preparation of Piezoelectric Material

**Figure 8:**
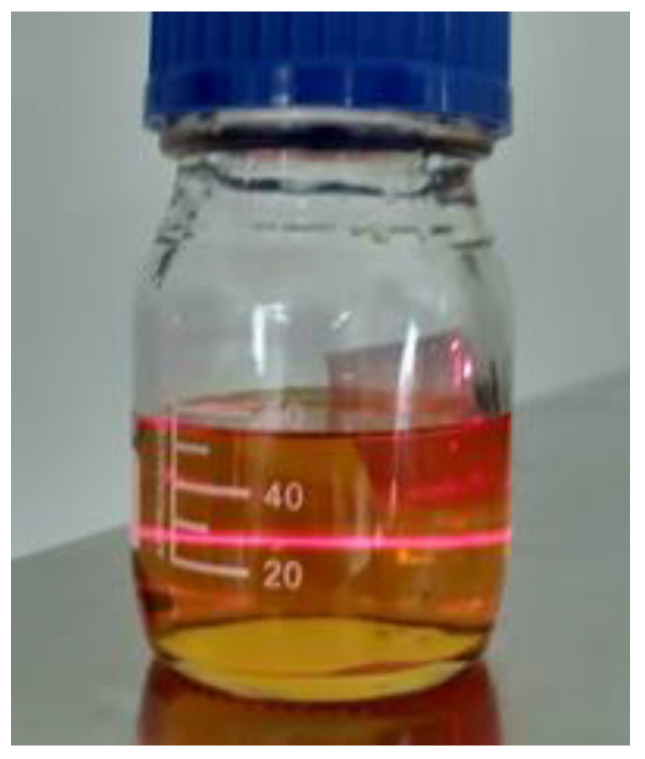
The lead zirconate titanate (PZT) sol.

Before fabricating the chip, we need to prepare the piezoelectric material. Lead zirconate titanate (PZT) is used as a piezoelectric material, which is prepared by sol–gel method [31,32]. In the preparation process of PZT sol, lead acetate trihydrate, zirconium nitrate and titanium butoxide are used as the metal ion sources of PZT. The 2-methoxyethanol is used as the solvent, acetylacetone is used as the stabilizer of the reaction, acetic acid is used as the catalyst while adjusting the pH during the reaction, and formamide is a drying agent. The molar ratio of Zr to Ti in PZT sol, the content of lead and the concentration of the solution will affect the piezoelectric properties of the final PZT film. In the preparation process, it is necessary to select appropriate parameters to obtain better piezoelectric performance. The prepared PZT sol is bright and light yellow, and the Tyndall effect will occur when there is a light beam passing through, as shown in Figure 8.

### 4.2. Fabrication of Microsensor Chip

**Figure 9:**
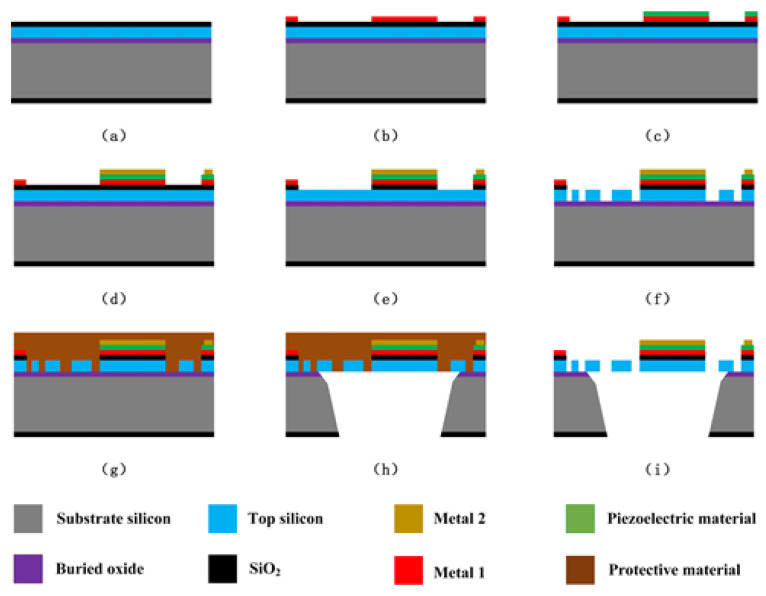
Main steps of the fabrication process. (**a**) Growing thermal SiO_2_. (**b**) Sputtering and patterning metal materials. (**c**) Depositing and patterning piezoelectric material. (**d**) Sputtering and patterning metal materials. (**e**) Patterning thermal SiO_2_. (**f**) Etching the top silicon layer. (**g**) Spin-coating protective materials. (**h**) Removing the oxides and etching the substrate silicon. (**i**) Remov-ing protective materials.

The proposed EFM is fabricated on a silicon-on-insulator (SOI) die. Because the thickness of sensing/shielding electrodes is 5 μm, we choose a SOI wafer with a 5-μm-thick top silicon layer, a 1-μm-thick buried oxide layer, and a 400-μm-thick substrate layer silicon. The main steps of the fabrication process are described as follows, as shown in Figure 9. 

(a) Growing thermal SiO_2_ of 500nm on the SOI wafer, SiO_2_ as an insulating layer. (b) Sputtering and patterning metal materials of Ti/Pt of 50/200 nm, and patterning metal by lift-off process with negative photoresist. (c) Depositing and patterning piezoelectric material, and patterning piezoelectric material by CHF_3_. (d) Sputtering and patterning metal materials of Au of 200 nm, and patterning metal by lift-off process with negative photoresist. (e) Patterning thermal SiO_2_ from the front side, and removing SiO_2_ by CHF_3_. (f) Etching the top silicon layer to form a structural layer by deep reactive ion etching (DRIE). (g) Spin-coating polyimide and photoresist on the front side as protective materials for substrate silicon etching. (h) Removing the oxides by CHF_3_, and etching the substrate silicon by DRIE to release the movable structure. (i) Removing protective materials by oxygen plasma.

**Figure 10:**
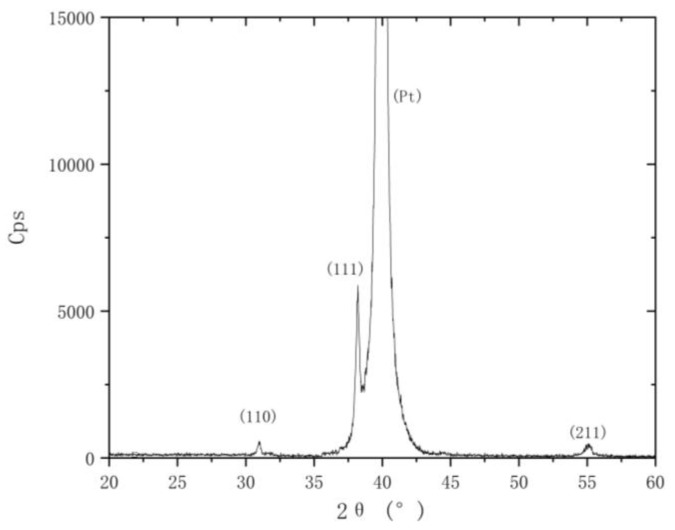
X-ray diffraction (XRD) of the PZT film. The crystal orientations of the PZT film along the (110), (111) and (211) directions have diffraction intensity, while there is almost no diffraction intensity at other diffraction angles; the PZT film has the highest diffraction intensity along the (111) direction.

Notably, fabricating the piezoelectric film and releasing the movable structure are necessary steps for the fabrication process. For the fabrication process of the piezoelectric film, firstly, the prepared PZT sol is spin-coated on the corresponding driving structure, then the amorphous film is obtained by low-temperature heat treatment, and finally, high-temperature thermal annealing is performed to form the piezoelectric driving film. The PZT film is analyzed by X-ray diffraction (XRD), as shown in Figure 10.

It can be seen from Figure 10 that the PZT film has completed the crystallization of the perovskite, the PZT film has the highest diffraction intensity along the (111) direction and the PZT film has good crystal orientation along the (111) direction.

In addition, etching the substrate silicon layer is a necessary step for releasing the movable structure. The protective materials are essential in protecting the structure on the silicon layer in etching the substrate silicon layer, so photoresist and polyimide are used together as protective materials. 

**Figure 11:**
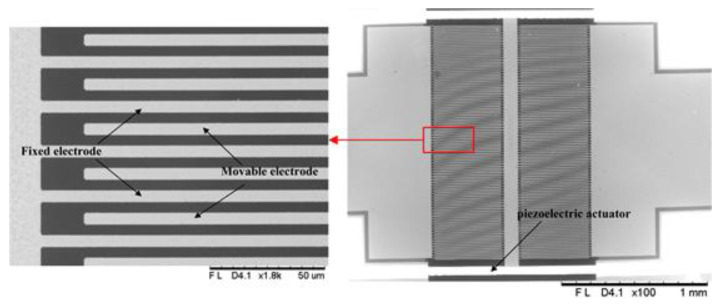
Scanning electron micrograph (SEM) photos of the microsensor chip. The widths of the fixed and movable electrodes are both 5 μm; the gap between the fixed and movable electrodes is 5 μm.

The scanning electron micrograph (SEM) photos of the fabricated microsensor chip are shown in Figure 11.

## 5. Conclusions

This paper proposes an electric field microsensor with mutual shielding electrodes. Electrodes in this EFM work as both sensing electrodes and shielding electrodes, and the movable electrodes are vertically driven in a piezoelectric way. Mutual shielding electrodes can double the sensing area of the EFM when compared with previous EFMs with grounded shielding electrodes, and ultimately improve the sensitivity of the sensor. The electrode structure adopts an interdigital structure, and the EFM has been simulated by finite element methods. Simulation results show that, since the sensing area of this EFM is doubled, the variation of induced charge is twice, and therefore the output signal of the sensor is increased. In this paper, the piezoelectric material PZT is prepared by the sol-gel method, and the piezoelectric film is fabricated. From the photo of XRD, it is analyzed that the PZT film has completed the crystallization of the perovskite.

## Figures and Tables

**Table 1:** The key parameters of the proposed EFM.

Structural Parameters	Value
width of fixed and movable electrodes	5 µm
gap between fixed and movable electrodes	5 µm
thickness of fixed and movable electrodes	5 µm
length of fixed and movable electrodes	500 µm
width of piezoelectric actuators	50 µm
thickness of piezoelectric actuators	0.6 µm
length of piezoelectric actuators	550 µm
number of piezoelectric actuators	4
number of fixed electrodes	84 × 2
number of movable electrodes	84 × 2

**Table 2:** Induced charge variations on electrodes.

d (μm)	ΔQ (× 10^−14^(C))	ΔQ_3_ (× 10^−14^(C))
−10	−9.59	4.78
−8	−8.8	4.37
−6	−7.56	3.74
−4	−5.7	2.81
−2	−3.11	1.53
0	0	0
2	3.17	−1.49
4	5.88	−2.73
6	7.94	−3.63
8	9.41	−4.23
10	10.41	−4.57

## References

[1] Luo F.S., Zhuang H.C., He Y.H., Zhang J., Chen M.J., Liu Y.M., Jiang D.Z., Qiu X.G., Chen Z.Y. (2000). The principle of micro-rocket electric field instrument and its application. Chin. J. Geophys..

[2] Tant P., Bolsens B., Sels T., Van Dommelen D., Driesen J., Belmans R. (2007). Design and application of a field mill as a high-voltage DC meter. IEEE Trans. Instrum. Meas..

[3] Montanya J., Bergas J., Hermoso B. (2004). Electric field measurements at ground level as a basis of lightning hazard warning. J. Electrost..

[4] Fort A., Mugnaini M., Vignoli V., Rocchi S., Perini F., Monari J., Schiaffino M., Fiocchi F. (2011). Design, modeling, and test of a system for atmospheric electric field measurement. IEEE Trans. Instrum. Meas..

[5] Winn W.P., Byerley L.G. (2006). Electric field growth in thunderclouds. Q. J. R. Meteorol. Soc..

[6] Parizotto R., Mesquita A., Porto R.W. Design and calibration of an atmospheric electric field sensor with wireless comunication. Proceedings of the 2015 International Symposium on Lightning Protection (XIII SIPDA).

[7] Michishita K., Ishii M., Hojo J.I. (1996). Measurement of horizontal electric fields associated with distant cloud-to-ground strokes. J. Geophys. Res. Atmos..

[8] Gerrard C.A., Gibson J.R., Jones G.R., Holt L. (1998). Measurements of power system voltages using remote electric field monitoring. IEE Proc. Gener. Transm. Distrib..

[9] Vaillancourt G.H., Carignan S., Jean C. (2002). Experience with the detection of faulty composite insulators on high-voltage power lines by the electric field measurement method. IEEE Trans. Power Deliv..

[10] Barthod C., Passard M., Bouillot J., Galez C., Farzaneh M. (2004). High electric field measurement and ice detection using a safe probe near power installations. Sens. Actuators Phys..

[11] Hornfeldt S.P. (1991). Dc-probes for electric field distribution measurements. Power Eng. Rev. IEEE.

[12] Hsu C.H., Muller R.S. Micromechanical electrostatic voltmeter. Proceedings of the 1991 International Conference on Solid-State Sensors and Actuators.

[13] Horenstein M.N., Stone P.R. (2001). A micro-aperture electrostatic field mill based on mems technology. J. Electrost..

[14] Riehl P.S., Scott K.L., Muller R.S., Howe R.T., Yasaitis J.A. (2003). Electrostatic charge and field sensors based on micromechanical resonators. J. Microelectromech. Syst..

[15] Roncin A., Shafai C., Swatek D.R. (2005). Electric field sensor using electrostatic force deflection of a micro-spring supported membrane. Sens. Actuators Phys..

[16] Chen X., Peng C., Tao H., Ye C., Bai Q., Chen S., Xia S. (2006). Thermally driven micro-electrostatic fieldmeter. Sens. Actuators Phys..

[17] Peng C., Chen X., Ye C., Tao H., Cui G., Bai Q., Chen S., Xia S. (2006). Design and testing of a micromechanical resonant electrostatic field sensor. J. Micromech. Microeng..

[18] Denison T., Jinbo K., Shafran J., Judy M., Lundberg K. A self-resonant mems-based electrostatic field sensor with 4v/m/hz sensitivity. Proceedings of the 2006 IEEE International Solid State Circuits Conference.

[19] Bahreyni B., Wijeweera G., Shafai C., Rajapakse A. (2008). Analysis and design of a micromachined electric-field sensor. J. Microelectromech. Syst..

[20] Takeshi K., Syoji O., Masaharu T., Ryutaro M., Toshihiro I. (2008). Microelectromechanical systems-based electrostatic field sensor using pb(zr,ti)o 3 thin films. Jpn. J. Appl. Phys..

[21] Kobayashi T., Oyama S., Okada H., Makimoto N., Tanaka K., Itoh T., Maeda R. An electrostatic field sensor driven by self-excited vibration of sensor/actuator integrated piezoelectric micro cantilever. Proceedings of the 2012 IEEE 25th International Conference on Micro Electro Mechanical Systems (MEMS).

[22] Yang P., Peng C., Zhang H., Liu S., Fang D., Xia S. A high sensitivity soi electric-field sensor with novel comb-shaped microelectrodes. Proceedings of the 2011 16th International Solid-State Sensors, Actuators and Microsystems Conference.

[23] Ghionea S., Smith G., Pulskamp J., Bedair S., Meyer C., Hull D. Mems electric-field sensor with lead zirconate titanate (pzt)-actuated electrodes. Proceedings of the 2013 IEEE sensors.

[24] Yang P., Peng C., Fang D., Wen X., Xia S. (2013). Design, fabrication and application of an SOI-based resonant electric field microsensor with coplanar comb-shaped electrodes. J. Micromech. Microeng..

[25] Chen T., Shafai C., Rajapakse A., Park B.Y. (2014). Micromachined electric field mill employing a vertical moving shutter. Procedia Eng..

[26] Feng K., Tong J., Wang Y., Fang D., Xia S. (2014). Electric field microsensor based on the structure of piezoelectric interdigitated cantilever beams. J. Electron. (China).

[27] Williams K.R., De Bruyker D.P., Limb S.J., Amendt E.M., Overland D.A. (2014). Vacuum steered-electron electric-field sensor. J. Microelectromech. Syst..

[28] Chen T., Shafai C., Rajapakse A., Liyanage J.S.H., Neusitzer T.D. (2016). Micromachined AC/DC electric field sensor with modulated sensitivity. Sens. Actuators Phys..

[29] Kainz A., Steiner H., Schalko J., Jachimowicz A., Kohl F., Stifter M., Beigelbeck R., Keplinger F., Hortschitz W. (2018). Distortion-free measurement of electric field strength with a MEMS sensor. Nat. Electron..

[30] Chu Z., Peng C., Ren R., Ling B., Zhang Z., Lei H., Xia S. (2018). A high sensitivity electric field microsensor based on torsional resonance. Sensors.

[31] Xiao M., Li S., Lei Z. (2015). Study of (111)-oriented PZT thin films prepared by a modified sol–gel method. J. Mater. Sci. Mater. Electron..

[32] Laishram R., Thakur O.P. (2014). Nanostructured PZT/PT multilayered thin films prepared by sol–gel process. Mater. Lett..

